# Comparing effects of beetroot juice and Mediterranean diet on liver enzymes and sonographic appearance in patients with non-alcoholic fatty liver disease: a randomized control trials

**DOI:** 10.3389/fnut.2023.1181706

**Published:** 2023-08-17

**Authors:** Hawal Lateef Fateh, Sameeah Abdulrahman Rashid, Sarmad S. Muhammad, Sabah H. Al-Jaf, Ayad M. Ali

**Affiliations:** ^1^Nursing Department, Kalar Technical College, Sulaimani Polytechnic University, Sulaymaniyah, Iraq; ^2^Nursing Department, Kalar Technical College, Garmian Polytechnic University, Kalar, Iraq; ^3^Department of Surgery, College of Medicine, Hawler Medical University, Erbil, Iraq; ^4^Medical Laboratory Technology Department, Kalar Technical College, Sulaimani Polytechnic University, Sulaymaniyah, Iraq; ^5^Medical Laboratory Technology Department, Kalar Technical College, Garmian Polytechnic University, Kalar, Iraq; ^6^Department of Chemistry, College of Science, University of Garmian, Sulaimani, Iraq

**Keywords:** mediterranean diet, beetroot juice, nonalcoholic fatty liver, RCT, NAFLD

## Abstract

**Background:**

In both developed and developing countries, non-alcoholic fatty liver disease (NAFLD) has lately risen to the top of the list of chronic liver illnesses. Although there is no permanent cure, early management, diagnosis, and treatment might lessen its effects. The purpose of conducting the current study is to compare the effects of beetroot juice and the Mediterranean diet on the lipid profile, level of liver enzymes, and liver sonography in patients with NAFLD.

**Methods:**

In this randomized controlled trial, 180 people with a mean age of (45.19 ± 14.94) years participated. Participants ranged in age from 19 to 73. The mean weight before intervention was (82.46 ± 5.97) kg, while the mean weight after intervention was roughly (77.88 ± 6.26) kg. The trial lasted for 12 weeks. The participants were split into four groups: control, a Mediterranean diet with beet juice (BJ + MeD), Mediterranean diet alone (MeD), and beetroot juice (BJ). The Mediterranean diet included fruits, vegetables, fish, poultry, and other lean meats (without skin), sources of omega-3 fatty acids, nuts, and legumes. Beetroot juice had 250 mg of beetroot. Data analysis was done using SPSS software (version 26.0). *p* < 0.05 is the statistical significance level.

**Results:**

Following the intervention, Serum Bilirubin, alkaline phosphatase (ALP), alanine transaminase (ALT), serum cholesterol (CHOL), triglyceride (TG), and low-density lipoprotein (LDL) levels were significantly decreased in the BJ + MeD, BJ, and MeD groups (*p* = 0.001). Also, high-density lipoprotein (HDL) significantly increased in the BJ + MeD, BJ, and MeD groups (*p* = 0.001), while decreasing in the Control group (*p* = 0.001).

**Conclusion:**

The research findings indicate a significant reduction in hepatic steatosis among the groups receiving beetroot juice (BJ) and beetroot juice combined with the Mediterranean diet (BJ + MeD). This suggests that beetroot juice holds potential as an effective treatment for non-alcoholic fatty liver disease (NAFLD) in adults. Furthermore, the combination of beetroot juice with the Mediterranean diet showed enhanced efficacy in addressing NAFLD.

**Clinical trial registration:** ClinicalTrials.gov, identifier NCT05909631.

## Introduction

Non-alcoholic fatty liver disease (NAFLD) has recently become the most common chronic liver disease in both industrialized and developing countries (1). It is characterized by the deposit of fat in the liver, which is not caused by alcohol consumption and is frequently linked to other metabolic diseases such as hyperlipidemia, cardiovascular disease, hepatocellular carcinoma, type 2 diabetes, and obesity (2–5). According to studies, NAFLD and its link to obesity will considerably increase liver-related morbidity and mortality by 2030 (6). The prevalence of NAFLD varies among different populations, with the Middle East having the highest rates (32% of the population is thought to be affected), followed by the United States and South America (30%). The estimated prevalence for other regions, including Asia, Europe, and Africa, ranges from 27 to 13% (7). As the prevalence of NAFLD rises, the search for effective and accessible treatment options becomes more important. The conventional management of NAFLD involves lifestyle modifications, including dietary changes and physical activity (8).

Recently, there has been an increase in interest in the efficacy of specific dietary components in the management of NAFLD, such as beetroot supplementation and the Mediterranean diet (MeD). Beetroot, which is high in bioactive compounds such as betaine, nitrates, and antioxidants, is one such supplement that has received attention for its potential health benefits (9). And also have a hepatoprotective effect and it effectively keeps away fat from depositing in the liver. This is probably due to the presence of betaine in beetroot which is a methyl group donor in the liver transmethylation process (10). Studies have examined the effects of beetroot supplementation on NAFLD, and the results have been encouraging (11). On the other hand, the Mediterranean diet (MeD) is defined as “a plant-based diet characterized by a high ratio of mono-unsaturated fatty acids (MUFA) to saturated fatty acids (SFA)” with a total fat accounting for 30–40% of daily energy consumption. The MeD is rich in olive oil, which is the main source of added fat together with nuts, has a high percentage of fibers, mainly obtained from vegetables, whole grains, and legumes, and is rich in fish and seafood, while meat and dairy products are consumed in a lower percentage. It is therefore a high-fat diet, with fat comprising 35–45% of the total energy intake, at least half of which should be from MUFAs. Carbohydrates constitute 35–40% and protein 15–20% of the energy intake (12).

According to research, the Mediterranean diet is good for several health outcomes, including liver function. Studies show that the MIND diet may improve liver health and reduce the risk of NAFLD (13–15).

Due to the continuous increase in the prevalence of NAFLD and its related risk factors in recent decades, the prevalence of unhealthy dietary patterns that follow them, we aimed to compare the effects of beetroot juice and the Mediterranean diet on lipid profile, level of liver enzymes and the liver sonography in patients with NAFLD.

## Method and materials

### Participants and study design

This Randomized Control Trial was conducted in Kalar City, Kurdistan region, Iraq. The Ethics Committee of Sulaimani Polytechnic University, Kalar technical college approved the study. It was also registered in ClinicalTrials.gov: (NCT05909631). Informed consent had been obtained from all participants before the start of the study and was approved by Sulaimany Polytechnic University, Kalar Technical College ethics committee, and the consent process was documented in their medical records.

All methods were carried out by relevant guidelines and regulations. All methods were carried out by relevant guidelines and regulations. This study was conducted by the Declaration of Helsinki.

Patients with NAFLD were identified and enrolled for this research at the PAR hospital in the Kurdistan Region of Iraq. Based on eligibility requirements, the participants were chosen by purposive sampling. Patients had to be between the ages of 19 and 73, and they had to have NAFLD as their main diagnosis. For a NAFLD diagnosis, ultrasound proof of fatty liver (stage I or above) and an elevated level of liver enzymes were required. An experienced radiologist did the ultrasonography. The grade of fatty liver was determined based on hepatic parenchymal brightness by ultrasound, and established methods were used to test the liver’s biochemical profile (16).

The following factors were used as exclusion criteria: a history of diseases like cardiovascular, liver, chronic hepatitis C, and kidney disease; supplement use within the previous 6 months; hormone therapy; use of drugs like amiodarone, methotrexate, and corticosteroids; cases of starvation or inadequate nutrition; pregnancy; metabolic disorders like Wilson disease and glycogen storage disease; a history of liver irradiation; and a refusal to continue the study protocol. We selected 180 participants for this study because they all met the requirements for inclusion. Finally, eligible participants were divided equally into four groups; a control group (*n* = 45) and three groups for the intervention group: *n* = 45 for beetroot juice plus Mediterranean diet (BJ + MeD), beetroot juice (BJ), and Mediterranean diet (MeD) via simple randomization. Then, the sequence of groups was drawn up by coin tossing.

For 12 weeks, for the group members who were receiving the MeD, we recommended consuming Mediterranean foods like fish, poultry, sources of omega-3, vegetables and colored fruits, legumes and nuts, and low-fat goods while avoiding inflammatory foods like high-fat foods, processed meats, fast food, simple sugars, chips, and soft drinks.

At baseline, the participants’ demographic and anthropometric information, including gender, age, weight, height, smoking status, location of residence, and physical activity, were recorded.

### Nutrients and bioactive compounds of beetroot

Beetroot is consisting of multiple biologically active phytochemicals including betalains (9) (e.g., betacyanins, and betaxanthins), flavonoids, polyphenols, Saponins (9) and inorganic Nitrate (NO3); it also a rich source of diverse minerals such as potassium, sodium, phosphorous, calcium, magnesium, copper, iron, zinc, and manganese (17). It is commonly consumed in the form of supplemental juice, powder, bread, gel, boiled, oven-dried, pickled, pureed, or jam-processed across different food cultures (18, 19). As shown in Table 1, 100 mL of beetroot juice is comprised of 95 Kcal energy, 22.6 g carbohydrates, 0.70 g proteins, 0.16 g total lipids, 0.91 g total dietary fiber, and 12 g total sugars. As such, the micro nutritional composition of 100 mL beetroot juice is estimated as 8.8 g sucrose, 0.86 g fructose, and 2.5 g glucose (9).

**Table 1:** Nutrient composition of beetroot and its byproducts (per 100 g or L).

	Raw	Cooked, boiled	Canned	Fresh juice
Water, g	87.58	87.6	90.96	–
Energy, kcal	43	44	31	30
Protein, g	1.61	1.68	0.91	1.02
Total fats, g	0.17	0.18	0.14	–
Carbohydrate, g	9.56	9.96	7.21	6.6
Fiber, g	2.8	2	1.8	0
Sugars, g	6.76	7.96	5.51	6.6
Calcium, mg	16	16	15	–
Iron, mg	0.8	0.79	1.82	0
Magnesium, mg	23	23	17	–
Phosphorus, mg	40	38	17	–
Potassium, mg	325	305	148	–
Sodium, mg	78	77	194	93
Zinc, mg	0.35	0.35	0.21	–
Vitamin C, mg	4.9	3.6	4.1	0
Thiamin, mg	0.031	0.027	0.01	–
Riboflavin, mg	0.04	0.04	0.04	–
Niacin, mg	0.334	0.331	0.157	–
Folate, μg	109	80	30	–
Total phenolic content^a^	255	238	192	225
Total flavonoid content^b^	260	261	173	126

a As mg gallic acid equivalent (GAE)/100 g.

b As mg rutin equivalent (RE)/100 g sample.

Moreover, various commercial organic and conventional beetroot juices, are reported to contain total sugar, vitamin C, and total flavonoids within a range of 1.73–7.85 g, 10.75–20.36 mg, and 2.02–2.36 mg (per 100 g), respectively (20). Betalains make up to ~70–100% of the phenolic composition of beetroot, limited to 0.8–1.3 g/L of fresh beetroot juice (about 60% betacyanins and 40% betaxanthins) (21).

Beetroot is classified as one of the ten plants with the highest antioxidant activity (9). It is believed to be the main commercial source of betalains, in concentrated forms, powder, or natural dyes in gelatins, confectionery, dairy, meat, and poultry-derived products (9). According to Baião et al., flavonoids change vegetable processing while polyphenols remain active after *in vitro* digestion, yet found in the highest ratio in beetroot gel than other conformations including beetroot juice (9).

### Dietary intake

To eliminate potential confounding variables, we matched the four groups according to sex, degree of physical activity, and caloric consumption. Participants conduct a 24 h dietary recall once a week to assess MeD compliance. Other groups received a 3 day food record questionnaire at the start and conclusion of the trial (1 day off and 2 days non-off). Using the Modified Nutritionist IV Software, the calorie, macronutrient, micronutrient, and water intakes were calculated (version 3.5.2, First Data-Bank; Hearst Corp., San Bruno, CA).

### Monitoring and follow-up

To control adherence to the MeD, patients complete a 24 h recall (weekly consumption data). Patients were also followed up every week by telephone to be aware of possible side effects and to ensure the use of beetroot juice.

### Nutritional intervention

250 mL of concentrated beetroot juice, 250 mL of a placebo (red carmoisine food color and a little quantity of a sweetener diluted in water), and beetroot juice were given to participants in the BJ groups and BJ + MeD groups (subjects swirled 250 mL of concentrated beetroot juice orally). This was carried out in the morning, 30 min before breakfast, and on an empty stomach.

### Fatty liver index

FLI was first presented by Bedogni et al. in 2006 with thirteen variables (including age, gender, ethanol intake, AST, ALT, GGT, WC, BMI, the sum of four skinfolds, Insulin, glucose, TG, and cholesterol), four of which remained as predictors in the equation (22, 23).


$$\begin{array}{c} \mathrm{FLI}=\left(e^{0.953*\mathrm{loge}\;\left(\mathrm{triglycerides}\right)+0.139*\mathrm{BMI}+0.718*\mathrm{loge}\;\left(\mathrm{ggt}\right)+0.053*\mathrm{waist circumference}-15.745}\right)/\\ \left(1+e^{0.953*\mathrm{loge}\;\left(\mathrm{triglycerides}\right)+0.139*\mathrm{BMI}+0.718*\mathrm{loge}\;\left(\mathrm{ggt}\right)+0.053*\mathrm{waist circumference}-15.745}\right)\\ \times 100. \end{array}$$

According to the area under the receiver operator characteristic curve (AUROC), the FLI’s fatty liver detection accuracy was 0.83 (95% CI: 0.825 to 0.842). The FLI scores are between 0 and 100. Thus, with a high degree of diagnostic accuracy, FLI scores of 30 and 60 indicated the absence or presence of fatty liver, respectively (22).

### Physical activity

The BEACK Questionnaire was used to assess the degree of physical activity among the patients (24) at the start of the investigation.

### Biochemical parameter measurements

After fasting for 10–12 h, 10 mL of whole blood was drawn from each participant at the beginning and end of the study. After centrifuging the samples, the serum was extracted. The serum was kept at −80°C for upcoming experiments. The levels of All biochemical investigations including liver function tests (ALT, AST, ALP, and S. Bilirubin), lipid profile tests (serum triglyceride (TG), cholesterol (Chol), cholesterol LDL and cholesterol HDL), and the grade of fatty liver were also assessed. Hence, COBASE C111 fully automated analyzer was used for analysis.

### Liver ultrasound

The ultrasound examination was performed by two experienced radiologists with more than 15 years of experience in the field. The machines used for the scanning were the GE Logiq7 and Philips HD11, both equipped with a 2–5 MHz curved probe.

The diagnosis of fatty liver was based on parenchymal brightness, liver-to-kidney contrast, bright vessel walls, gallbladder wall definition, and diaphragmatic definition (25).

The fatty disease was graded and labeled as mild, moderate, or severe, or grades 0 to 3 (with 0 being normal) based on the degree of parenchymal brightness.

**Figure 1:**
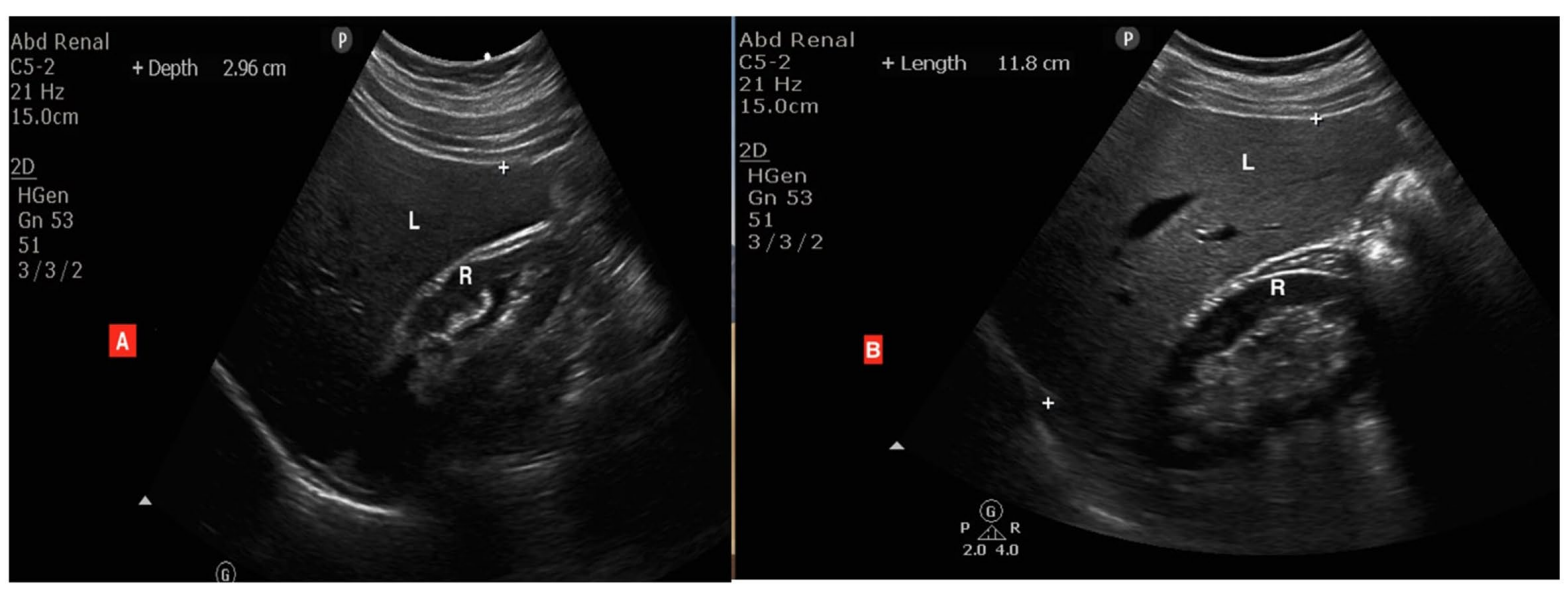
Ultrasound images of two different cases: **(A)** a normal liver with similar brightness of the liver (L) and renal cortex (R), while **(B)** is a patient with grade 1 (mild) fatty changes showing the liver (L) is brighter than the renal cortex (R) but the wall of the intrahepatic vessels is still clearly seen.

**Grade 0 (normal liver)**: the liver and renal cortex are of similar echogenicity (Figure 1A).

**Grade 1 (mild)**: slight diffuse increase in the fine echoes of liver parenchyma with appreciable periportal and diaphragmatic echogenicity (Figure 1B).

**Grade 2 (moderate)**: moderately diffuse increase in fine echoes of the liver parenchyma obscuring periportal echogenicity, but diaphragmatic echogenicity is still appreciable.

**Grade 3 (severe)**: marked increase in fine echoes of liver parenchyma with poor or no visualization of the intrahepatic vessel borders, diaphragm, and posterior portion of the right lobe of the liver.

When the discrepancy between the grades of fatty liver developed between the two radiologists, the final decision was made by consensus.

### Data analysis

SPSS software (version 26.0) was used to analyze the data, also data were presented as means and standard deviations for continuous variables and frequencies, and Categorical data were expressed as counts and percentages. The Kolmogorov–Smirnov test and histogram were applied to ensure the normal distribution of variables. Chi-square was used for the sociodemographic characteristics of the subjects and differences between qualitative. Paired *t*-test and one-way analysis of variance (ANOVA) were applied to determine differences between continuous variables. Statistical significance was considered when *p* < 0.05 was used.

## Results

### Participants’ characteristics

**Figure 2:**
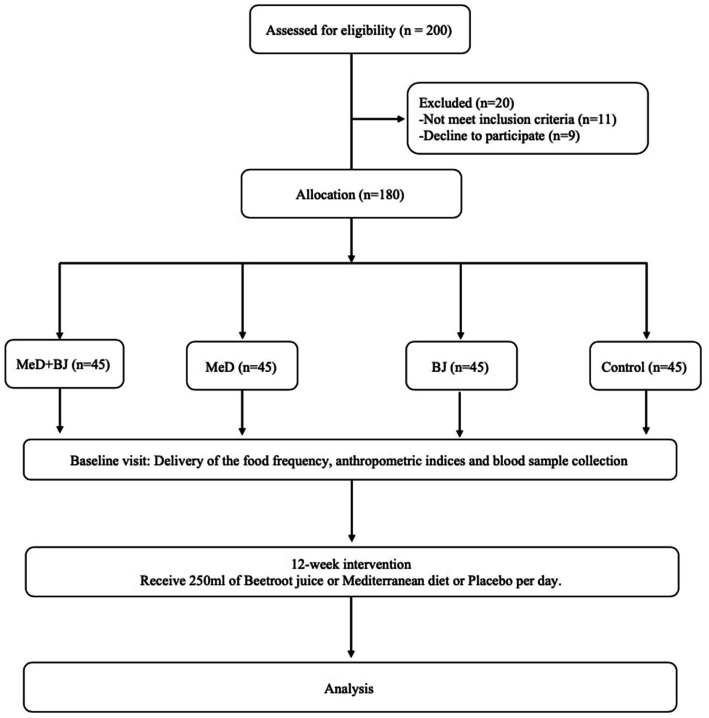
Follow chart of the study. Med plus BJ; Med; BJ and Control.

A total of 180 participants of different ages were enrolled in the current study and were randomly assigned to one of the four groups (total = 180 cases; *n* = 45). According to the following chart in Figure 2, all participants completed the study, and their data were entered into the final analysis. Sociodemographic characteristics are minimized in Table 2. As shown in Table 2, no age differences are observed between the groups. There was no difference in BMI between the groups at the beginning of the study, but after the study, statical differences were observed in each group before and after the study.

**Table 2:** Baseline characteristics in the four groups.

Variables		BJ + MeD	MeD	BJ	Control	*p value
		Mean ± SD/*n* (%)				
Age (year)		44.49 ± 15.73	47.31 ± 15.75	44.91 ± 15.24	44.04 ± 13.2	0.736
Gender *n* (%)	Male	17 (9.4)	23 (12.8)	28 (15.6)	28 (15.6)	0.062
	Female	28 (15.6)	22 (12.2)	17 (9.4)	17 (9.4)	
Smoker *n* (%)	No	40 (22.2)	35 (19.4)	33 (18.3)	28 (15.6)	0.031
	Yes	5 (2.8)	10 (5.6)	12 (6.7)	17 (99.4)	
Physical activity (Met h/day)	Light	8 (4.4)	17 (9.4)	14 (7.8)	15 (8.3)	0.516
	Moderate	22 (12.2)	16 (8.9)	16 (8.9)	17 (9.4)	
	High	15 (8.3)	12 (6.7)	15 (8.3)	13 (7.2)	
Living Place *n* (%)	Rural	21 (11.7)	21 (11.7)	14 (7.8)	22 (12.2)	0.294
	Urban	24 (13.3)	24 (13.3)	31 (17.2)	23 (12.8)	
BMI kg/m^2^	Before	29.96 ± 2.77	29.19 ± 3.94	28.01 ± 2.55	28.62 ± 3.84	0.042
	After	27.30 ± 3.45	27.75 ± 3.74	26.46 ± 2.56	27.82 ± 3.36	0.191
	**p value	<0.001	<0.001	<0.001	0.037	
Waist circumference (cm)	Before	90.25 ± 5.74	91.34 ± 4.53	91.72 ± 6.99	92.33 ± 4.89	0.354
	After	87.59 ± 5.14	89.91 ± 4.77	90.17 ± 6.69	91.54 ± 4.89	0.008
	**p value	<0.001	<0.001	<0.001	0.032	

Data presented as Mean ± SD/*n* (%), *obtained by Chi square test and **obtained by paired *t*-test. BJ + MeD, beetroot juice and Mediterranean diet; MeD, Mediterranean diet; BJ, beetroot juice.

### Liver enzymes

Table 3 shows the results for the liver enzyme test at the end of the study were as follows; T.S.B. in the intervention groups was significantly decreased as compared with that of the control group (*p* < 0.001). Further, a comparison of the intergroup revealed that at the end of the current study, the T.S.B. in groups BJ + MED (*p* < 0.001), and BJ (*p* < 0.001) in comparison with the beginning of the study, had decreased significantly. Furthermore, no significant differences in serum AST levels were found between the intervened groups and the control group at the end of the study, whereas the intergroup comparison for serum AST levels revealed that at the end of the current study, the amount of serum AST in the BJ (p.001) and MED (*p* < 0.001) groups was significantly lower than at the start of the study. In the case of ALT, there is a significant decrease in the concentration of ALT in the intervened groups as compared with that of the control group (*p* < 0.001). Further, a comparison of the intergroup data revealed that the concentration of serum ALT at the end of the study was significantly lower in the MED (*p* < 0.001) and BJ + MED (*p* < 0.001) groups. Finally, the results for serum ALP concentration showed that the concentration of serum ALP at the end of the study in the intervention groups was significantly lower in comparison with that of the control group. Besides, the intergroup comparison for serum ALP at the beginning and end of the study revealed a significant decrease in serum ALP concentration in the BJ + MED (*p* < 0.001) and BJ (*p* < 0.001) groups at the end of the current study.

**Table 3:** Comparisons of the changes from baseline to the end of the intervention for biochemical parameters.

Variables		BJ + MeD	MeD	BJ	Control	**p value
		Mean ± SD				
Liver Enzymes						
S. Bilirubin	Before	1.46 ± 0.3	1.27 ± 0.4	1.15 ± 0.2	1.44 ± 0.3	<0.001
	After	0.87 ± 0.1	1.16 ± 0.2	0.72 ± 0.1	1.50 ± 0.3	<0.001
	*p value	<0.001	0.155	<0.001	0.431	
AST	Before	63.53 ± 8.80	61.32 ± 7.79	61.43 ± 8.85	63.85 ± 8.78	0.349
	After	59.16 ± 7.34	58.63 ± 7.87	58.18 ± 6.33	61.11 ± 6.93	0.014
	*p value	0.150	<0.001	<0.001	0.101	
ALT	Before	46.75 ± 6.61	44.44 ± 7.18	37.63 ± 3.45	46.89 ± 5.71	<0.001
	After	42.87 ± 6.50	38.06 ± 7.30	36.61 ± 5.87	44.50 ± 6.56	<0.001
	*p value	0.010	<0.001	0.320	0.059	
ALP	Before	121.8 ± 5.7	119.3 ± 6.5	119.0 ± 8.5	120.2 ± 3.1	<0.001
	After	114.8 ± 6.8	117.4 ± 6.3	113.6 ± 7.6	122.0 ± 3.6	<0.001
	*p value	<0.001	0.316	<0.001	<0.001	
Lipid Profile						
S.TG	Before	243.1 ± 14.3	240.4 ± 13.3	233.5 ± 14.1	173.8 ± 12.6	<0.001
	After	175.5 ± 10.6	217.9 ± 10.6	217.6 ± 11.6	175.7 ± 14.6	<0.001
	*p value	<0.001	<0.001	<0.001	0.494	
S. Cholesterol	Before	244.9 ± 28.8	232.5 ± 19.4	228.9 ± 6.2	224.6 ± 16.8	<0.001
	After	188.8 ± 15.8	219.6 ± 5.3	210.5 ± 3.7	221.1 ± 16.7	<0.001
	*p value	<0.001	<0.001	<0.001	0.304	
LDL	Before	142.4 ± 9.2	143.5 ± 11.0	140.2 ± 9.2	147.1 ± 11.2	<0.001
	After	128.8 ± 6.3	130.7 ± 5.4	132.2 ± 5.8	135.7 ± 9.7	<0.001
	*p value	<0.001	<0.001	<0.001	<0.001	
HDL	Before	30.2 ± 5.1	33.1 ± 4.8	29.8 ± 3.9	30.9 ± 3.9	<0.001
	After	41.5 ± 3.2	39.8 ± 3.8	36.6 ± 4.2	28.8 ± 4.01	<0.001
	*p value	<0.001	<0.001	<0.001	<0.001	

Data presented as Mean ± SD, *p* value obtained by *Paired *t*-test and **One-way Anova. BJ + MeD, beetroot juice and Mediterranean diet; MeD, Mediterranean diet; BJ, beetroot juice.

### Lipid profile

The assessment of the lipid profile at the end of the study was as follows: the results for serum TG concentration in the intervention groups were significantly reduced in comparison with that of the control group (*p* < 0.001). Further, the intragroup comparison revealed that at the end of the current study, the concentrations of serum TG in the BJ + MED (*p* < 0.001), MED (*p* < 0.001), and BJ (*p* < 0.001) groups were significantly decreased in comparison with the beginning of sample collection. Further, serum cholesterol concentrations in the intervention groups were significantly reduced in comparison with those of the control group (*p* < 0.001). In addition, the intergroup comparison revealed that serum cholesterol concentrations in the BJ + MED (*p* < 0.001), MED (*p* < 0.001), and BJ (*p* < 0.001) groups had been significantly reduced at the end of the current study. Besides, the concentration of serum LDL in the intervention groups was significantly decreased at the end of the study as compared with that of the control group (*p* < 0.001), and the intragroup comparison revealed that the concentration of serum LDL in all four groups was significantly reduced at the end of the study (*p* < 0.001). Finally, there were significant differences in serum HDL concentration between the intervention groups and the control group at the end of the study. However, as shown in the intragroup comparison, we notice that the concentration of serum HDL in the BJ + MED (*p* < 0.001), MED (*p* < 0.001), and BJ (*p* < 0.001) groups was significantly increased in Table 3.

### Nutritional assessment

Structural and cross-sectional questionnaires were carried out for all groups regarding the baseline at the end of the intervention for Mediterranean diet components. Significant differences were reported between groups in variables within vegetables, legumes, nuts and seeds, low-fat dairy, red and processed meats, poultry and fish, and MUFA/SFA (*p* < 0.001) while no significant differences were reported in other variables: total cereal, fermented dairy, and fruits (Table 4). Additionally, significant changes (*p* < 0.001) were reported within groups regarding total cereal, low-fat dairy, and MUFA/SFA in the control group, fermented dairy legumes, nuts, and seeds; low-fat dairy, red and processed meats, poultry, fish; and fruits in the BJ + Med, group; and red and processed meats, fish, and MUFA/SFA in the MeD group, while only MUFA/SFA was reported as a significant variable in the BJ group. According to daily energy intake, at the beginning of the study, the highest daily energy intake was for the BJ + Med group (2,278 ± 76.37 kcal/d), and the lowest was the control group (2,116 ± 59.28 kcal/d). At the end of the study, there was a change in the daily energy intake of all groups, with the greatest amount of change in the BJ + Med group.

**Table 4:** Comparisons of the changes from baseline to the end of the intervention for Mediterranean diet components.

Variables		BJ + MeD	MeD	BJ	Control	**p value
		Mean ± SD				
Daily energy intake (kcal/day)	Before	2,278 ± 76.37	2,143 ± 35.14	2,199 ± 62.63	2,116 ± 59.28	<0.001
	After	2096 ± 24.19	2,127 ± 32.75	2,152 ± 32.94	2,102 ± 52.13	<0.001
	*p value	<0.001	0.019	<0.001	0.765	
MeD component						
Vegetables (g/d)	Before	307.6 ± 118.1	308 ± 104.8	315.5 ± 109.1	296.4 ± 130.9	0.891
	After	366.6 ± 145 ± 7	329.3 ± 126.2	320.6 ± 116.4	248.3 ± 113.4	<0.001
	*p value	0.053	0.424	0.810	0.106	
Total cereal (g/d)	Before	295.5 ± 83.6	297.2 ± 90.9	286.3 ± 70.3	316.9 ± 80.0	0.341
	After	313.6 ± 92.8	295.2 ± 101.1	293.0 ± 64.1	264.4 ± 80.5	0.059
	*p value	0.307	0.922	0.629	<0.001	
Fermented dairy (g/d)	Before	452.8 ± 76.1	442.8 ± 89.6	461.3 ± 87.9	459.1 ± 84.9	0.732
	After	497.4 ± 97.8	460.4 ± 83.6	475.9 ± 90.8	458.4 ± 77.4	0.131
	*p value	<0.001	0.337	0.457	0.969	
Legume, nut and seed (g/d)	Before	38.6 ± 20.3	38.4 ± 15.4	36.7 ± 18.2	40.0 ± 17.4	0.854
	After	60.1 ± 23.0	39.2 ± 22.9	38.2 ± 16.4	371 ± 14.4	<0.001
	*p value	<0.001	0.834	0.661	0.374	
Low fat dairy(g/d)	Before	127.9 ± 40.6	121.1 ± 40.8	110.9 ± 51.4	127.0 ± 25.7	0.068
	After	106.6 ± 37.9	111.2 ± 39.4	104.9 ± 44.3	90.9 ± 55.6	<0.001
	*p value	<0.001	0.262	0.589	<0.001	
Red and processed meats (g/d)	Before	34.5 ± 15.5	26.7 ± 16.8	41.6 ± 16.7	37.4 ± 17.5	<0.001
	After	24.9 ± 14.2	19.1 ± 10.8	36.8 ± 17.7	33.8 ± 13.4	<0.001
	*p value	<0.001	<0.001	0.175	0.271	
Poultry (g/d)	Before	109.3 ± 44.4	110 ± 39.9	98 ± 46.1	118 ± 48.4	0.214
	After	137.7 ± 48.9	120.5 ± 41.4	108.6 ± 43.9	117.3 ± 45.0	<0.001
	*p value	<0.001	0.243	0.248	0.946	
Fish (g/d)	Before	36.7 ± 9.46	34.6 ± 7.4	37.1 ± 9.3	37.1 ± 10.8	0.542
	After	47 ± 9.6	42.7 ± 10.9	39.5 ± 10.9	39.3 ± 9.5	<0.001
	*p value	<0.001	<0.001	0.333	0.317	
Fruits (g/d)	Before	302.9 ± 56.1	295.3 ± 54.6	308.5 ± 51.6	289.8 ± 56.3	0.385
	After	329.6 ± 66.3	305.9 ± 67.7	301.6 ± 60.6	296.3 ± 60.7	0.072
	*p value	<0.001	0.444	0.575	0.613	
MUFA/SFA (g/d)	Before	0.78 ± 0.13	0.71 ± 0.12	0.90 ± 0.11	0.91 ± 0.17	<0.001
	After	0.90 ± 0.17	0.86 ± 0.11	0.92 ± 0.13	0.79 ± 0.17	<0.001
	*p value	0.382	<0.001	<0.001	<0.001	

Data presented as Mean ± SD, *p* value obtained by *Paired *t*-test and **One-way Anova. BJ + MeD, beetroot juice and Mediterranean diet; MeD, Mediterranean diet; BJ, beetroot juice.

### Fatty liver status assessment

**Figure 3:**
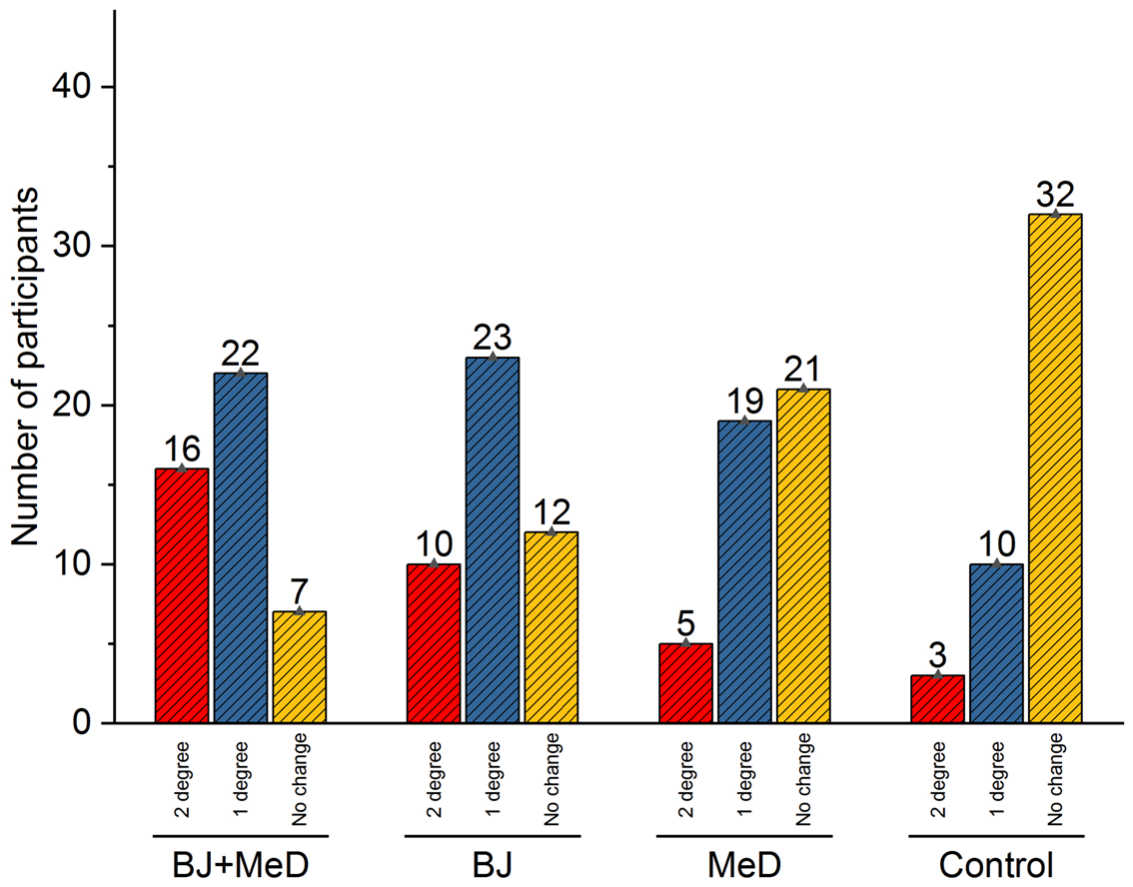
Fatty liver grade change after at the end of intervention.

Currently, our results show a significant decrease in fatty liver accumulation. Especially in the BJ + MeD and BJ groups during the study. This reduction was reported at a higher level in the BJ + MeD group (one-degree reduction: 48%; and 2-degree decreasing: 35%, while in the BJ group, one-degree decreasing: 51%; and 2-degree decreasing: 22%, also, 71% of the control group showed no change in their liver fat accumulation; Figure 3).

**Figure 4:**
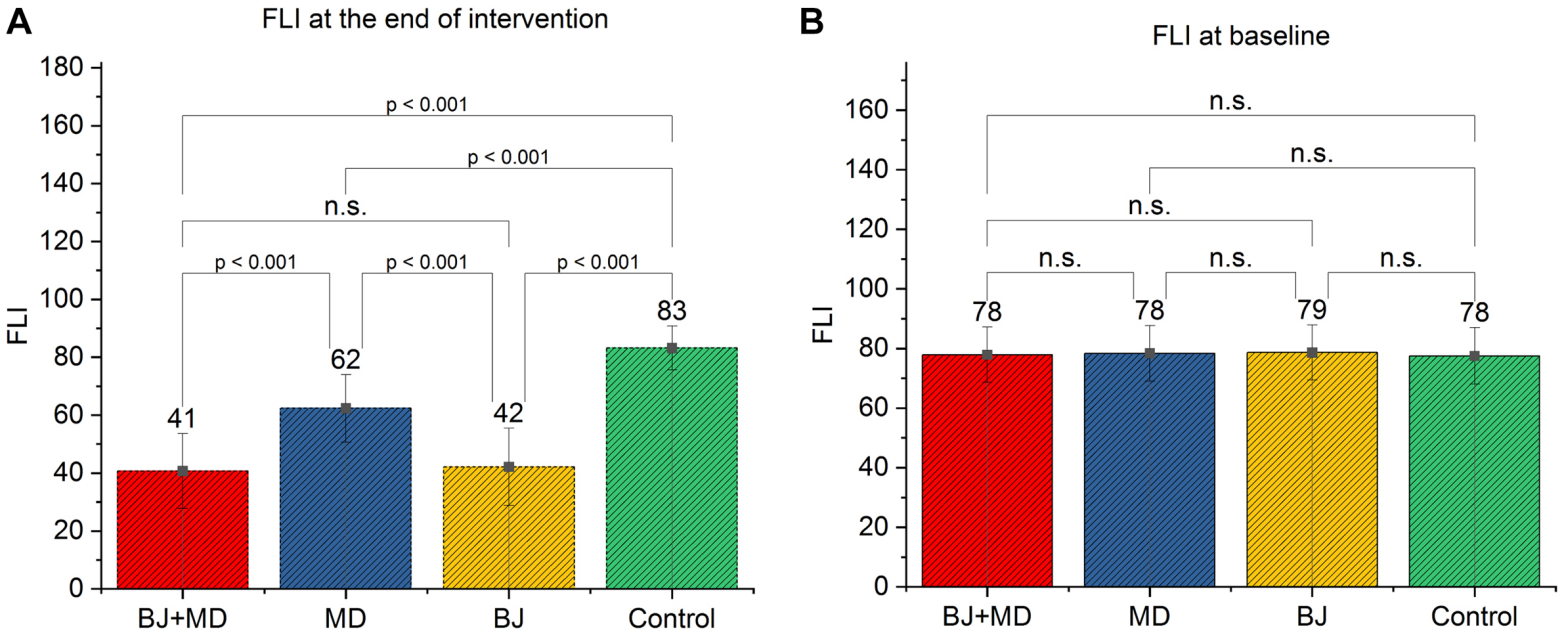
FLI of study participants according to groups: **(A)** FLI at the end of intervention, while **(B)** FLI at the baseline of intervention.

As shown in Figure 4, the FLI of study participants according to groups Figure 4A, is the beginning of the study and there is no difference between the FLIs of the participants and all participants have high FLIs. However, Figure 4B shows the end of our study and tells us that the BJ + MD group had a significantly lower FLI. There was a difference between all groups before and after the study.

## Discussion

To our knowledge, in this area, this is the first randomized controlled trial observing the comparing Effects of Beetroot juice and the Mediterranean diet on the level of liver enzymes and sonography in those cases with NAFLD. We found that consumption of 250 mL of red beetroot juice for 12 weeks, parallel to the Mediterranean diet, had a significant beneficial impact on some liver enzymes and lipid profiles (S. Bilirubin, ALT, ALP, TG, Cholesterol, LDL, and HDL). Flavonoids, dietary nitrate, and Saponin are the three main components of beetroot (26). Saponin was previously reported to be a significant contributor to lowering cholesterol activity (27) and dietary nitrate, which can increase the production of nitric oxide in the body. Nitric oxide has been shown to have beneficial effects on the liver, including reducing inflammation and improving blood flow (28). Also, fibers are one of the major components of beetroot and the Mediterranean diet. Insoluble fibers can decrease the level of lipid profile since they do not absorb in the intestine, so they can bind to the cholesterol and remove it from the body Soluble fibers have been shown to increase the rate of bile excretion, therefore reducing serum total and LDL cholesterol (27).

In this study, a significant decrease in BMI was observed in the BJ, BJ + MeD, and MeD groups after 12 weeks of intervention. Likewise, a meta-analysis including seven observational reports and six randomized clinical trials revealed that adherence to MeD can significantly decrease BMI (effect size = −1.23 kg/m^2^; 95% CI − 2.38 to −0.09 kg/m^2^), weight (effect size = −4.13 kg; 95% CI − 8.06 to −0.20 kg) (29).

Our results showed that the level of TC, TG, LDL-C, and HDL-C after intervention was reduced in the BJ + MeD group compared to the BJ or MeD groups. Both beetroots juice and the Mediterranean diet have been studied for their effects on lipid profiles, specifically serum triglyceride (TG) concentrations. In the current study, serum TG levels were significantly affected. Furthermore, the intergroup comparison revealed that at the end of the study, serum TG concentrations in the BJ + MeD, MeD, and BJ groups were significantly lower than at the start of sample collection within daily feed intake. Besides, serum cholesterol concentrations in the intervention groups were significantly lower than in the control group. A cohort study by Baratta et al. revealed that adherence to MeD had a preventive effect on NAFLD (OR: 0.801, *p* = 0.018) (30). Kaliora et al. (2019) shows that adherence to the Med diet can reduce the concentration of serum triglycerides (effect size = −33.01 mg/dL; 95% CI − 52.84 to −13.18 mg/dL), and the total cholesterol (effect size = −6.89 mg/dL; 95% CI − 14.90 to 1.12 mg/dL), which can be theoretically translated to protective effects in NAFLD (31).

A 2022 published review found that drinking beetroot juice for 4 weeks significantly reduced serum TG concentrations in healthy adults (32). In a conducted study by de Castro et al. (33), Dyslipidemia overweight subjects used freeze-dried red beet leaves (2.8 g) for 4 weeks, which resulted in lower LDL-C, especially in intervention groups but not in control groups. Another study revealed that female soccer players reported a significant reduction in the concentration of LDL-C in the test group who consumed 200 mL of beetroot juice 2 h before their training (34). However, consuming powder beetroot for 12 weeks reduced the level of lipid profile and liver enzymes. Amnah and Alushaibani (2013) reported in their study that, when done on rats that consume biscuits prepared with beetroot powder, they can significantly reduce liver enzymes, cholesterol, and total lipids in cases compared to controls (35). Nouri et al. (2017) observed that male Wistar rats with liver illnesses had lower liver enzyme levels after consuming beetroot juice (36).

Combining beetroot juice and the Mediterranean diet may have even greater effects on lipid profiles. A 2020 study published in the Journal of Functional Foods found that combining beetroot juice and the Mediterranean diet for 12 weeks significantly reduced serum TG concentrations in overweight and obese adults (37). Sigh et al. (2015) conducted a human study that revealed that consuming beetroot juice reduced the lipid profile, including LDL, triglycerides, and total cholesterol levels, while also significantly increasing HDL levels in physically active individuals (38).

Hence, our finding showed that the bilirubin level in participants in the intervention groups was considerably lower than in the control groups. Additionally, the total bilirubin level in groups BJ + MeD and BJ had considerably decreased. Several studies have found that beetroot juice can improve liver enzyme levels in people with liver disease. For example, a 2017 study published in the World Journal of Gastroenterology found that drinking beetroot juice for 12 weeks significantly reduced levels of liver enzymes in people with non-alcoholic fatty liver disease (39).

Moreover, there were no discernible differences in serum AST levels between the baseline and end of the intervention in the BJ + MeD group. However, an intragroup comparison of AST showed that the amounts of AST in the BJ and MeD groups had significantly decreased from the study’s start to the end. When compared to the control group, the percentage of ALT in the intervention groups significantly decreased.

Integration of BJ the key liver disease biomarkers, AST and ALP, was considerably reduced in the NAFLD patients’ treatment regimen compared to the standard of care. Because NAFLD patients have greater degrees of fibrosis and increased AST (40), a possible strategy to stop the advancement of liver fibrosis is to enhance AST. The interaction between time and groups showed that BJ’s influence on ALT increased over time, even though BJ could not have a significant impact on ALT in this study. Future research should assess BJ’s impact on ALT over a longer period than 6 months. In a different study, the hepatoprotective effects of beetroot juice on liver damage in male Sprague Dawley rats were assessed. It was discovered that beetroot juice had dose-dependent hepatoprotective effects on liver damage (41). This finding supports our hypothesis about the discrepancy between our results and the study by Srivastava and colleagues. Moreover, Ozsoy-Sacan et al. (42) in their study assessed the *Beta vulgaris* extract effect on the liver of diabetic rats and found that the level of ALT, AST, and ALP significantly reduced in the intervened group, as compared to the placebo or control group.

On NAFLD, MeD, which primarily consists of higher intakes of fruits, vegetables, whole-grain cereals, plant-based proteins (legumes and nuts), micronutrients like potassium, calcium, and vitamin C, dietary fiber, omega-3 fatty acids, and monounsaturated fatty acids, as well as polyphenols and other antioxidant agents, has been linked to several beneficial outcomes (43). Currently, strong evidence-based supports the advantages of healthy dietary patterns in controlling most of the risk factors for NAFLD (44); for instance, Dorosty et al. reported that consuming whole grains for 12 weeks, independent of weight loss, beneficially affected concentration of liver enzymes, and fatty liver in the patients with NAFLD (45).

Consuming a healthy diet that contains sufficient amounts of vegetables and fruits (about 400 g/day) prevents the incidence of non-communicable diseases (30). Because it is high in fiber, antioxidants, and essential minerals, beetroot juice is one of the best homemade remedies for fatty liver. Its nutrients help to detoxify the liver and improve the processes of fat elimination. In mammals, the main sources of nitrate are diet (particularly leafy green vegetables) and endogenous synthesis (46). According to a new study co-authored by Harvard T.H. Chan School of Public Health researchers, a Mediterranean diet that includes more green plant matter may cut the risk of non-alcoholic fatty liver disease in half (14).

The main finding of the current study was a significant reduction in liver steatosis in BJ and BJ + MeD groups. It was also observed that this reduction in hepatic steatosis in the group members receiving Beetroot juice was increasingly more significant than that in the Mediterranean diet group. The results of this study were supported by Srivastava et al. (2019) (34). And these effects may be due to the presence of dietary nitrate or antioxidant properties.

Finally, based on the comparison between the intervention groups in this study, it was concluded that beetroot juice (BJ group) is more effective than the Mediterranean diet (MeD group) in the treatment of NAFLD. By contrast, taking the Mediterranean diet (MeD group) and beetroot juice (BJ group) together (BJ + MeD group) is even more effective than taking either beetroot juice or the Mediterranean diet alone. The strength of the current study is that it is the first to estimate the effect of two intervened groups alone and together on the NAFLD. However, there are some limitations, such as the lack of complete adherence to a diet by some participants.

## Conclusion

Red beetroot stands out as one of the most nutritionally dense foods found in the plant kingdom, containing a wealth of essential nutrients such as vitamins, minerals, phenols, carotenoids, nitrate, ascorbic acids, and betalains. Notably, both beetroot juice and the Mediterranean diet have demonstrated promising advantages for liver well-being. Nevertheless, additional research is necessary to comprehensively grasp their impact on liver enzyme levels and liver sonography. Future studies must examine any potential adverse effects that may arise from the consumption of beetroot juice.

## Data availability statement

The raw data supporting the conclusions of this article will be made available by the authors, without undue reservation.

## Ethics statement

The studies involving humans were approved by Kalar technical college ethic commite. The studies were conducted in accordance with the local legislation and institutional requirements. The participants provided their written informed consent to participate in this study.

## Author contributions

HF designed the study and analyzed the data. HF, SR, SM, SA-J, and AA prepared the draft of the manuscript. All authors contributed to the article and approved the submitted version.

## Funding

This research was supported by Garmian Polytechnic University.

## Conflict of interest

The authors declare that the research was conducted in the absence of any commercial or financial relationships that could be construed as a potential conflict of interest.

## Publisher’s note

All claims expressed in this article are solely those of the authors and do not necessarily represent those of their affiliated organizations, or those of the publisher, the editors and the reviewers. Any product that may be evaluated in this article, or claim that may be made by its manufacturer, is not guaranteed or endorsed by the publisher.
